# Interaction of vitamin D with membrane-based signaling pathways

**DOI:** 10.3389/fphys.2014.00060

**Published:** 2014-02-18

**Authors:** María Jesús Larriba, José Manuel González-Sancho, Félix Bonilla, Alberto Muñoz

**Affiliations:** ^1^Instituto de Investigaciones Biomédicas “Alberto Sols,” Consejo Superior de Investigaciones Científicas, Universidad Autónoma de MadridMadrid, Spain; ^2^Department of Medical Oncology, Hospital Universitario Puerta de Hierro MajadahondaMajadahonda, Spain

**Keywords:** 1α,25(OH)_2_D_3_, VDR, membrane-based signaling, Wnt, growth factors, cytokines, paracrine effects

## Abstract

Many studies in different biological systems have revealed that 1α,25-dihydroxyvitamin D_3_ (1α,25(OH)_2_D_3_) modulates signaling pathways triggered at the plasma membrane by agents such as Wnt, transforming growth factor (TGF)-β, epidermal growth factor (EGF), and others. In addition, 1α,25(OH)_2_D_3_ may affect gene expression by paracrine mechanisms that involve the regulation of cytokine or growth factor secretion by neighboring cells. Moreover, post-transcriptional and post-translational effects of 1α,25(OH)_2_D_3_ add to or overlap with its classical modulation of gene transcription rate. Together, these findings show that vitamin D receptor (VDR) cannot be considered only as a nuclear-acting, ligand-modulated transcription factor that binds to and controls the transcription of target genes. Instead, available data support the view that much of the complex biological activity of 1α,25(OH)_2_D_3_ resides in its capacity to interact with membrane-based signaling pathways and to modulate the expression and secretion of paracrine factors. Therefore, we propose that future research in the vitamin D field should focus on the interplay between 1α,25(OH)_2_D_3_ and agents that act at the plasma membrane, and on the analysis of intercellular communication. Global analyses such as RNA-Seq, transcriptomic arrays, and genome-wide ChIP are expected to dissect the interactions at the gene and molecular levels.

## Introduction

The active vitamin D metabolite 1α,25-dihydroxyvitamin D_3_ (1α,25(OH)_2_D_3_) is a key regulator of gene expression in higher organisms. It modulates the activity of the vitamin D receptor (VDR), a member of the superfamily of nuclear hormone receptors that regulate gene transcription. Genome-wide chromatin immunoprecipitation studies have shown that VDR binds to hundreds of genome sites even in the absence of 1α,25(OH)_2_D_3_ and that ligand binding increases and partially changes these binding sites, which depend on the cell type and the duration of treatment (Carlberg and Campbell, 2013). While a subset of VDR binding sites may be responsible for the control of gene expression (VDREs or vitamin D response elements), others might be temporary anchorage places for a population of unliganded “dormant” VDR. According to the classical view, VDR binds DNA as heterodimers with a retinoid X receptor (RXRα, β, or γ) and, upon ligand binding, changes the transcription rate of neighboring genes.

However, many genes whose expression is altered by 1α,25(OH)_2_D_3_ do not contain VDREs. Putative mechanisms of this action include post-transcriptional regulation via changes in the levels of microRNAs that modulate the half-life and/or translation of their messenger RNAs (Thorne et al., 2011; Wang et al., 2011; Alvarez-Díaz et al., 2012; Kasiappan et al., 2012; Guan et al., 2013). Also, 1α,25(OH)_2_D_3_ may regulate genes post-translationally via changes in the phosphorylation or other modifications of proteins which affect their stability (Lin et al., 2003; Li et al., 2004), or through changes in the level or activity of proteases that target them (Alvarez-Díaz et al., 2010).

Increasing importance has recently been accorded to another mechanism of 1α,25(OH)_2_D_3_ action: the modulation of signaling pathways triggered by other agents at the plasma membrane. Indeed, a number of studies have shown that 1α,25(OH)_2_D_3_ modulates the effects of growth factors and cytokines by altering either their cytosolic signaling pathways or the activity of target transcription factors in the nucleus, or even in a paracrine fashion by inhibiting their synthesis and secretion by neighboring cells.

Here we review the available data on these non-classical, alternative mechanisms by which 1α,25(OH)_2_D_3_ modulates gene expression. Notably, for specific genes such as c-*MYC*, both direct transcriptional and indirect modes of regulation by 1α,25(OH)_2_D_3_ have been described (Pan and Simpson, 1999; Pálmer et al., 2001; Toropainen et al., 2010; Salehi-Tabar et al., 2012).

## Interaction of 1α,25(OH)_2_D_3_ with Wnt, hedgehog, and notch pathways

Wnt, Hedgehog, and Notch signaling pathways, which have long been known to play crucial roles during development, are now considered critical for many tumorigenic processes in which they function abnormally due to mutation and/or changes in expression of components.

**Figure 1:**
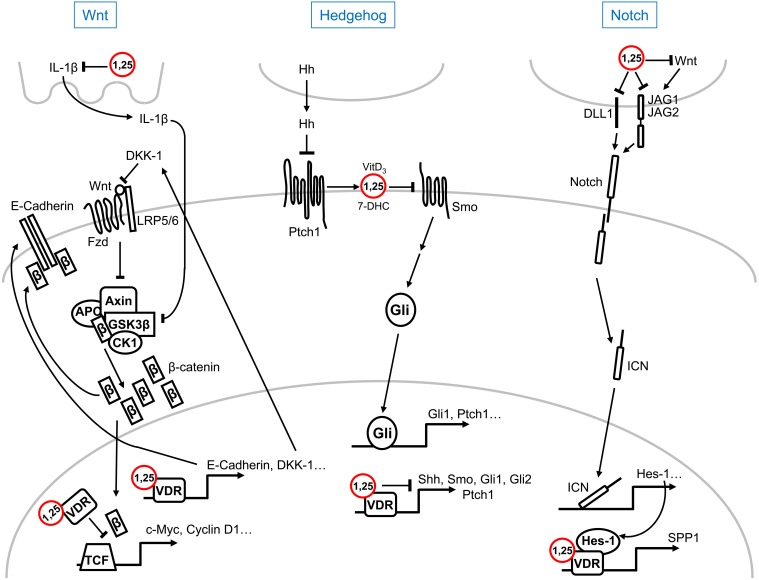
**Schematic representation of the multilevel crosstalk of 1α,25(OH)_2_D_3_ (1,25) with Wnt, Hedgehog, and Notch signaling pathways**. For simplicity, only main components and regulators of the pathways are shown. Explanations, details, and references can be found in the text.

Wnt factors activate several signaling pathways upon binding to different plasma membrane receptors: the canonical or Wnt/β-catenin and the non-canonical (planar polarity, Ca^2+^…) pathways (Clevers and Nusse, 2012). Activation of the Wnt/β-catenin pathway by mutation of *APC* or *AXIN* tumor suppressor genes or of *CTNNB1*/β-catenin oncogene together with changes in the expression of a number of regulatory genes (*SFRPs*, *DICKKOPF (DKK)s*…) is a hallmark of most colorectal cancers and of a variable proportion of several other malignancies (Clevers and Nusse, 2012). A series of studies report that 1α,25(OH)_2_D_3_ antagonizes Wnt/β-catenin signaling in colon cancer cells by several mechanisms: the reduction of transcriptionally active β-catenin/T-cell factor complexes, the induction of β-catenin relocation from the nucleus toward the *adherens junctions* structures at the plasma membrane, and the increase in the level of the Wnt inhibitor DKK-1 (Pálmer et al., 2001; Shah et al., 2006; Aguilera et al., 2007) (Figure 1). In this way, the pathway endpoint, i.e., the activation of β-catenin target genes, is attenuated by 1α,25(OH)_2_D_3_ (Pálmer et al., 2001). Emphasizing the importance of this action, an additional indirect mechanism of Wnt/β-catenin antagonism in colon cancer has been proposed involving IL-1β, which will be reviewed in section 1α,25(OH)_2_D_3_ and Cytokines. Although 1,25(OH)_2_D_3_ inhibits β-catenin/TCF transcriptional activity in colon and other cancer cells, the upregulation of the Wnt/β-catenin pathway by either ligand-activated or unliganded VDR has been described in osteoblasts and keratinocytes, where it promotes bone formation and hair follicle differentiation, respectively (Larriba et al., 2013). However, the results reported in keratinocytes are controversial: while VDR enhances Wnt signaling through direct binding to Lymphocyte Enhancer-binding Factor (LEF)-1 independently of ligand and β-catenin (Luderer et al., 2011), ligand-activated VDR is believed to inhibit Wnt/β-catenin signaling (Bikle, 2011; Jiang et al., 2012).

Inhibition of Hedgehog (Hh) signaling by vitamin D compounds has also been suggested. In a study combining experiments in zebrafish, the yeast *Pichia pastori* and mouse fibroblasts, secreted vitamin D_3_, or its precursor 7-dehydrocholesterol (7-DHC), was shown to mediate the paracrine inhibition of Smoothened (Smo) by Patched (Ptch)1 which leads to pathway inactivation (Bijlsma et al., 2006). In the model proposed, which includes the binding of vitamin D_3_ to Smo at high (micromolar) concentrations, Hh ligands activate the pathway by blocking the induction of the secretion of vitamin D_3_/7-DHC by Ptch1 (Bijlsma et al., 2006) (Figure 1). The Hh pathway is aberrantly activated in basal cell carcinoma, the most frequent human tumor type. Interestingly, 1α,25(OH)_2_D_3_ inhibits proliferation and induces differentiation of mouse basal cell carcinomas and embryonal rhabdomyosarcomas with an activated Hh pathway due to *Ptch1* deletion (Uhmann et al., 2011, 2012). As in the previous study, 1α,25(OH)_2_D_3_ acts at the level of Smo in a VDR-independent manner (Figure 1). Curiously, Tang et al. found that vitamin D_3_ inhibits Hh and cell proliferation more effectively than 7-DHC, 25(OH)D_3_, or 1α,25(OH)_2_D_3_ in murine basal cell carcinoma cells (Tang et al., 2011). Vitamin D_3_ also inhibits proliferation and Hh pathway through inactivation of Smo in cultured mouse pancreatic adenocarcinoma cells, but has no antitumor activity *in vivo* (Brüggemann et al., 2010). A common concern in all these studies is the high concentration of vitamin D_3_ required to observe the reported effects. Research in *Vdr*-deficient mice and in mouse skin explants has shown that lack of VDR increases the expression of several components of the Hh pathway such as *Shh*, *Smo*, *Gli1*, *Gli2*, and *Ptch1*, while 1α,25(OH)_2_D_3_ suppresses their expression (Bikle et al., 2013) (Figure 1). However, the interaction between 1α,25(OH)_2_D_3_ and Hh signaling in human skin remains to be elucidated.

Few studies link vitamin D with Notch signaling. Differentiation of human osteoblasts with vitamin D_3_ and dexamethasone distinctly affects the expression of Notch receptor family members (Schnabel et al., 2002). In rodent osteoblasts, the transcription factor Hes-1, which is an effector of the Notch pathway, enhances the induction of *SPP1*/osteopontin transcription by 1α,25(OH)_2_D_3_, indicating the collaboration of 1α,25(OH)_2_D_3_ and Notch pathways in bone remodeling (Shen and Christakos, 2005) (Figure 1). Transcriptomic analyses in human RWPE1 immortalized non-tumorigenic prostate cells showed the reduction of the RNA levels of the NOTCH ligands *JAGGED* (*JAG)1*, *JAG2*, and *Delta-like* (*DLL)1* by 1α,25(OH)_2_D_3_ (Kovalenko et al., 2010) (Figure 1). By contrast, no changes in the expression of *NOTCH-1* and *JAG1* were detected in cultured human keratinocytes upon 1α,25(OH)_2_D_3_ treatment (Reichrath and Reichrath, 2012). As *JAG1* transcription and, consequently, Notch signaling are upregulated by Wnt/β-catenin in colorectal cancer cells (Rodilla et al., 2009), the repressive effect of 1α,25(OH)_2_D_3_ on the Notch pathway in this system may be secondary to the antagonism of the Wnt/β-catenin pathway (Figure 1).

## Interplay of 1α,25(OH)_2_D_3_ with agents that trigger signaling pathways via plasma membrane kinase receptors

There is mutual antagonism between 1α,25(OH)_2_D_3_ and epidermal growth factor (EGF), a potent mitogen, in primary colon epithelial cells and in established colon (Caco-2) and breast (T47D) tumor cell lines. This is based on the cross-inhibition of the expression of their respective receptors, VDR and EGFR (Tong et al., 1998, 1999). However, it is a cell type-dependent effect as EGF increases VDR in the rat small intestine and 1α,25(OH)_2_D_3_ increases EGFR in BT-20 breast cancer cells (Bruns et al., 1989; Desprez et al., 1991). In addition, 1α,25(OH)_2_D_3_ inhibits EGFR signaling by increasing the level of E-cadherin protein at the plasma membrane, which downregulates EGFR (Pálmer et al., 2001; Andl and Rustgi, 2005), and by decreasing that of SPROUTY-2, a cytosolic protein that reduces EGFR ubiquitination, internalization and degradation (Cabrita and Christofori, 2008; Barbáchano et al., 2010).

Transforming growth factor (TGF)-β has opposite roles in carcinogenesis: it inhibits proliferation of normal epithelial cells, but it later induces epithelial-mesenchymal transition, immunosuppression and metastasis (Pickup et al., 2013). 1α,25(OH)_2_D_3_ induces the expression of the type I TGF-β receptor and both agents, 1α,25(OH)_2_D_3_ and TGF-β, cooperate in Caco-2 cell growth inhibition (Chen et al., 2002; Pálmer et al., 2003). Moreover, Smad3, a mediator of TGF-β signaling, is a co-activator of VDR and contributes to gene regulation by 1α,25(OH)_2_D_3_ (Yanagisawa et al., 1999), an effect that is abrogated by Smad7 in transfected COS-7 cells (Yanagi et al., 1999). Reinforcing the interaction between both signaling pathways, 1α,25(OH)_2_D_3_ induces the expression of Smad anchor for receptor activation (SARA) (Pálmer et al., 2003), which maintains the epithelial phenotype by recruiting Smads 2/3 to the activated TGF-β receptors and regulates endocytic trafficking of EGFR and other proteins (Tang et al., 2011; Kostaras et al., 2013). Notably, a recent study of R. M. Evans' group has revealed a genome-wide overlap of VDR and Smad3 binding sites that is responsible for the abrogation by VDR ligands of the TGF-β1-mediated activation of hepatic stellate cells during liver fibrosis (Ding et al., 2013). These authors show that TGF-β1 signaling redistributes VDR-binding sites in the genome and facilitates VDR binding at Smad3 profibrotic target genes. Upon ligand activation, VDR binding at coregulated genes decreases Smad3 occupancy at these sites, causing inhibition of fibrosis (Ding et al., 2013). This is a regulatory feed-back mechanism in which VDR ligands limit the fibrotic process and so ensure an appropriate non-pathological tissue response. Given the crucial roles of TGF-β in carcinogenesis, future studies should examine whether vitamin D compounds play similar roles in the maintenance of epithelial integrity opposing the onset of carcinomas.

1α,25(OH)_2_D_3_ and TGF-β interact also in bone. Curiously, in rat (UMR 106 and ROS 17/2.8) and human (MG-63) osteoblastic cells TGF-β increases VDR expression but inhibits the stimulation of osteocalcin and osteopontin transcription and RNA levels by 1α,25(OH)_2_D_3_ (Staal et al., 1994). TGF-β exerts this inhibitory effect by reducing the binding of VDR-RXR complexes to VDREs localized in the promoter of these genes without affecting the nuclear availability of VDR at least in ROS 17/2.8 cells (Staal et al., 1996). In contrast to the stimulation of osteocalcin synthesis in human and rat cells, 1α,25(OH)_2_D_3_ decreases osteocalcin production in mouse fetal long bone cultures and neonatal osteoblastic MC3T3 cells while stimulating bone resorption (Staal et al., 1998). This bone resorption action of 1α,25(OH)_2_D_3_ is dose-dependently inhibited by TGF-β (Staal et al., 1998).

A complex, cell type-, context- and sometimes age-dependent relation exists between 1α,25(OH)_2_D_3_ and insulin-like growth factors (IGF)-I and II. For instance, in C2C12 myoblasts 1α,25(OH)_2_D_3_ decreases IGF-I expression while it increases that of IGF-II (Garcia et al., 2011). In HT29 colon carcinoma cells several vitamin D compounds inhibit the secretion of IGF-II thus attenuating its cell proliferation activity (Oh et al., 2001). In addition, 1α,25(OH)_2_D_3_ blocks the mitogenic activity of insulin and IGF-I in MCF7 breast cancer cells, at least in part due to the inhibition of c-FOS upregulation (Vink-van Wijngaarden et al., 1996). 1α,25(OH)_2_D_3_ and IGF-I have also opposite effects on mouse long bones: IGF-I increases osteocalcin production, which is completely blocked by 1α,25(OH)_2_D_3_, and inhibits the enhancement of bone resorption caused by 1α,25(OH)_2_D_3_ (Staal et al., 1998). Furthermore, 1α,25(OH)_2_D_3_ variably regulates the expression of several IGF binding proteins (IGFBPs), a group of molecules with pleiotropic actions that transport IGFs and also modulate cell survival/apoptosis: 1α,25(OH)_2_D_3_ induces IGFBP3 expression in SW480-ADH colon carcinoma, SaOS-2 osteosarcoma, PC3 prostate cancer, MCF7 breast carcinoma and MCF-10A normal mammary cells (Pálmer et al., 2003; Matilainen et al., 2005; Malinen et al., 2011; Brosseau et al., 2013), IGFBP6 in SaOS-2, SW480-ADH and colon carcinoma HT29 cells (Oh et al., 2001; Pálmer et al., 2003; Matilainen et al., 2005), IGFBP1 and IGFBP5 in SaOS-2 and PC3 cells, and IGFBP4 in SaOS-2 cells (Matilainen et al., 2005). Conversely, 1α,25(OH)_2_D_3_ represses IGFBP4 in HT29 and SW480-ADH cells, and IGFBP2 in HT29 cells (Oh et al., 2001; Pálmer et al., 2003). In ovarian cells, 1α,25(OH)_2_D_3_ alone induces IGFBP1 production but, conversely, it enhances the inhibitory effect of insulin (Parikh et al., 2010). Curiously, recent studies show that IGFBP3 interacts with VDR (Li et al., 2013) and that IGFBP6 binds VDR and blocks the induction of osteoblast differentiation by 1α,25(OH)_2_D_3_ (Cui et al., 2011).

Cell type-dependent effects of 1α,25(OH)_2_D_3_ have also been described for hepatocyte growth factor (HGF) signaling. 1α,25(OH)_2_D_3_ activates the *HGF* gene promoter and induces HGF expression and secretion in rat NRK-49F renal interstitial fibroblasts (Li et al., 2005) and in human keloid fibroblasts (Zhang et al., 2011). Consistently with these results, vitamin D deficiency reduces HGF and HGF receptor/c-Met expression during liver regeneration in rats (Goupil et al., 1997). Conversely, 1α,25(OH)_2_D_3_ decreases the level of *HGF* RNA in human HL-60 promyelocitic leukemia cells (Inaba et al., 1993), smooth muscle cells (Shalhoub et al., 2010) and MG-63 osteosarcoma cells (Chattopadhyay et al., 2003). Moreover, the expression of c-Met is inhibited by 1α,25(OH)_2_D_3_ in human MHCC97 hepatocellular cell line (Wu et al., 2007). Curiously, 1α,25(OH)_2_D_3_ and HGF cooperate to increase osteogenic differentiation of human bone marrow stem cells and maturation of chondrocyte progenitor cells (Grumbles et al., 1996; D'Ippolito et al., 2002; Chen et al., 2011, 2012). Also, 1α,25(OH)_2_D_3_ and HGF additively inhibit proliferation of androgen-unresponsive prostate cancer cells (Qadan et al., 2000).

In concordance with its regulatory role in the organism, 1α,25(OH)_2_D_3_ favors physiological and homeostatic angiogenesis but inhibits angiogenesis in pathological conditions. Thus, 1α,25(OH)_2_D_3_ promotes myogenic differentiation of C2C12 cells by increasing the expression of two key angiogenic factors: vascular endothelial growth factor (VEGF) and fibroblast growth factor-1 (Garcia et al., 2013). In addition, 1α,25(OH)_2_D_3_ stimulates pro-angiogenic properties of endothelial progenitor cells by increasing VEGF levels (Grundmann et al., 2012). 1α,25(OH)_2_D_3_ also upregulates VEGF expression in osteoblast-like cells but not in breast cancer cells (Schlaeppi et al., 1997). Likewise, ED-71, a vitamin D analog, enhances VEGF expression and promotes angiogenesis in a murine bone marrow ablation model (Okuda et al., 2007). Indeed, increased production of VEGF in vascular smooth muscle cells results from the activation of a VDRE present in the *VEGF* gene promoter (Cardus et al., 2009). By contrast, 1α,25(OH)_2_D_3_ downregulates hypoxia-inducible factor (HIF)-1 and VEGF protein expression in several human colon, prostate and breast cancer cell lines (Ben-Shoshan et al., 2007), decreases VEGF production by human lumbar annulus cells (Gruber et al., 2008), and protects against diabetic retinopathy in rats by inhibiting VEGF expression in the retina (Ren et al., 2012).

1α,25(OH)_2_D_3_ also modulates the activity of signaling pathways mediated by other types of plasma membrane receptors such us G protein-coupled receptors. Shen et al. found that 1α,25(OH)_2_D_3_ suppresses the expression of parathyroid hormone-related protein (PTHrP) in prostate cancer cells via a negative VDRE localized within the non-coding region of the gene, thus antagonizing the induction of cell proliferation and of the expression of the pro-invasive integrin α_6_β_4_ exerted by PTHrP signaling (Shen et al., 2007).

## 1α,25(OH)_2_D_3_ and cytokines

The anti-inflammatory and immunomodulatory actions, and thus some of the anticancer and antimicrobial effects of 1α,25(OH)_2_D_3_, are mediated by the regulation of cytokine production and/or through the control of their receptors or downstream signaling pathways. Globally, 1α,25(OH)_2_D_3_ contributes to the autocrine and paracrine control of innate and adaptative immune responses (Adorini and Penna, 2008).

1α,25(OH)_2_D_3_ regulates the function of antigen-presenting cells and T-lymphocytes. It inhibits Th1 cells differentiation and, therefore, the secretion of Th1-type cytokines, enhances the development of Th2 cells, and induces tolerogenic monocytes and dendritic cells. IL-4 and IL-10 are among the commonly increased cytokines, while IL-1, IL-2, IL-6, IL-17, tumor necrosis factor (TNF)-α and interferon (IFN)-γ are decreased (Adorini and Penna, 2008).

Mechanistically, ligand-activated VDR directly downregulates the expression of IL-10, IL-2, and IL-12B in lipopolysaccharide-treated human monocytes (THP-1) through its binding to VDREs located in the genomic regions of these genes and the recruitment of the co-repressor NCOR/SMRT and histone deacetylases (Matilainen et al., 2010a,b; Gynther et al., 2011). Remarkably, IL-10 is downregulated by short 1α,25(OH)_2_D_3_ treatment (8 h) but upregulated at late time points (48 h) (Matilainen et al., 2010a). In addition, direct VDR binding to a single VDRE mediates the upregulation of *IL-8* gene by 1α,25(OH)_2_D_3_ in undifferentiated and differentiated THP-1 cells (Ryynänen and Carlberg, 2013).

1α,25(OH)_2_D_3_ also changes the expression of target genes in immune cells by repressing crucial transcription factors such as nuclear factor *kappa* B (NFkB) and signaling pathways such as Janus kinase-signal transducer and activator of transcription (JAK-STAT) (Yu et al., 1995; Muthian et al., 2006; Geldmeyer-Hilt et al., 2011). 1α,25(OH)_2_D_3_ also represses NFκB activity in fibroblasts and adipocytes (Harant et al., 1998; Mutt et al., 2012), and fibroblasts lacking VDR have increased NFκB activity (Sun et al., 2006). Direct (increase in IκBα expression and reduction of nuclear translocation of p65) and indirect (upregulation of IGFBP3 and clusterin) mechanisms contribute to the inhibition of NFκB activation (Krishnan and Feldman, 2010). D. Feldman's group has reported that, in addition to inhibiting NFκB, the anti-inflammatory effects of 1α,25(OH)_2_D_3_ in prostate cancer cells include the reduction of pro-inflammatory prostaglandins (PG) production via suppression of ciclooxygenase-2, downregulation of PG receptors, and upregulation of 15-hydroxyprostaglandin dehydrogenase, which inactivates PGs (Krishnan and Feldman, 2010). Moreover, 1α,25(OH)_2_D_3_ decreases the synthesis of pro-inflammatory IL-6 through the inactivation of p38 kinase due to the upregulation of the mitogen kinase phosphatase (MKP)5 and the blockade of TNF-α (Krishnan and Feldman, 2010). In Jurkat cells, the repression of *IL-2* gene by 1α,25(OH)_2_D_3_ is at least partially due to the blockade of NFATp/AP-1 complex formation at a positive regulatory NFAT-1 site, which is bound by VDR-RXR heterodimers (Alroy et al., 1995).

1α,25(OH)_2_D_3_ reduces the secretion of interleukin (IL)1-β in THP macrophages by blocking the activation of STAT1 (Kaler et al., 2009). As IL1-β activates the Wnt/β-catenin pathway in colon carcinoma cells via inhibition of GSK3β activity and subsequent stabilization and nuclear translocation of β-catenin, this mechanism may contribute to the antagonism of Wnt signaling by 1α,25(OH)_2_D_3_ (Kaler et al., 2009) (Figure 1). Curiously, IL-1α is believed to be upregulated and to mediate the antiproliferative effects of 1α,25(OH)_2_D_3_ in prostate progenitor/stem cells (Maund et al., 2011).

In human osteoblasts, 1α,25(OH)_2_D_3_ completely overrules the inhibitory effect of IFN-β on mineralization. This dominant effect on osteoblast differentiation and bone formation is reflected in the downregulation of IFN-related and -regulated genes by 1α,25(OH)_2_D_3_ (Woeckel et al., 2012). Concomitantly, 1α,25(OH)_2_D_3_ also induces activin A, a strong inhibitor of mineralization, and represses follistatin, the natural antagonist of activin A, to ensure a fine-tuned regulation of the mineralization process (Woeckel et al., 2013b).

Recent findings have underscored the complexity of 1α,25(OH)_2_D_3_ action and its role in the antimicrobial response as part of innate and adaptative immunity. Thus, activation of macrophage Toll-like receptors (TLRs) by intracellular bacteria such as *Mycobacterium tuberculosis* upregulates *VDR* and *CYP27B1* genes that allow the induction of the antimicrobial peptide cathelicidin by 1α,25(OH)_2_D_3_ (Liu et al., 2006). In monocytes, TLR activation triggers induction of defensin β4 (*DEFB4*) gene requiring the cooperation between IL-1β and 1α,25(OH)_2_D_3_, which is explained by the presence of one VDRE and two IL-1β-activatable NFkB sites in the *DEFB4* promoter (Liu et al., 2009). In addition, 1α,25(OH)_2_D_3_ is required for the antimicrobial effect of IFN-γ in human macrophages (Fabri et al., 2011). Moreover, by inducing the expression of TLR2 and CD14 receptors and cathelicidin, 1α,25(OH)_2_D_3_ mediates the effect of TGF-β favoring the response to microbial infection and wound injury by keratinocytes (Schauber et al., 2007). These findings show also unexpected cooperation of 1α,25(OH)_2_D_3_ with agents (IL-1β, TGF-β, IFN-γ) that are antagonistic in other cell types.

## Interplay of 1α,25(OH)_2_D_3_/VDR with transcription factors

Liganded or unliganded VDR interacts with or regulates the expression of a number of transcription factors that are downstream effectors of different signaling pathways (Table 1). An interesting example is the upregulation by 1α,25(OH)_2_D_3_ of *CDKN1B*/p27^Kip1^, a cell cycle regulator gene which lacks VDREs. 1α,25(OH)_2_D_3_ was first shown to induce *CDKN1B* transcription by stimulating the binding of Sp1 and NF-Y transcription factors to the *CDKN1B* promoter in the myelomonocytic U937 cell line (Inoue et al., 1999). Later, direct VDR-Sp1 interaction at the promoter Sp1 sites was described as responsible for this effect (Huang et al., 2004). In addition to the enhancement of transcription, 1α,25(OH)_2_D_3_ increases the stability of p27^Kip1^ protein by repressing p45^Skp2^, an F-box protein, through the induction of VDR-Sp1 complexes that together with histone deacetylase 1 are recruited to Sp1 sites at the p45^Skp2^ gene promoter (Lin et al., 2003; Li et al., 2004; Huang and Hung, 2006).

**Table 1:** **Interplay between VDR and other transcription factors**.

**Transcription factor**	**Biological effect**	**References**
Sp1/NF-Y	Potentiation	Inoue et al., 1999; Huang et al., 2004
AP-1/NFAT1	Repression	Towers et al., 1999
AP-1	Activation	Chen et al., 1999
CREB	Repression	Yuan et al., 2007
FOXO3a, FOXO4	Activation	An et al., 2010
p53	Mutual repression	Stambolsky et al., 2010; Chen et al., 2013
PPAR-α/δ	Activation	Sertznig et al., 2009a,b
PPAR-γ	Variable	Alimirah et al., 2012; Woeckel et al., 2013a
RAR	Variable	Jiménez-Lara and Aranda, 1999; Tavera-Mendoza et al., 2006; Anand et al., 2008; Ng et al., 2010
ER	Downregulation	Krishnan et al., 2010; Swami et al., 2013
AR	Crossregulation	Zhao et al., 1999; Ting et al., 2005
PIT-1	Downregulation	Seoane and Pérez-Fernández, 2006

The granulocyte-macrophage colony-stimulating factor (*GM-CSF*) gene is another example of unusual regulation by 1α,25(OH)_2_D_3_. Ligand-activated VDR represses *GM-CSF* through a composite DNA element recognized by Jun-Fos heterodimers (AP-1) and nuclear factor of activated T-cells (NFAT)1 (Towers et al., 1999). In the absence of RXR, VDR binds to c-Jun and stabilizes AP-1 bound to DNA, which outcompetes NFAT1 and decreases *GM-CSF* transcription. In Caco-2 cells, 1α,25(OH)_2_D_3_ stimulates AP-1 via activation of protein kinase C-α, ERK and JNK leading to cell differentiation (Chen et al., 1999).

In renal cells, 1α,25(OH)_2_D_3_ suppresses renin gene expression by blocking the cyclic AMP response element (CRE) through direct binding of VDR to CRE-binding protein (CREB) and so, inhibiting the binding of CREB to the CRE (Yuan et al., 2007). By a complex mechanism, 1α,25(OH)_2_D_3_ also regulates several Forkhead box (FOX) transcription factors. Ligand-activated VDR binds FOXO3a and FOXO4 together with their regulators, sirtuin 1 deacetylase and protein phosphatase 1, inducing deacetylation and dephosphorylation of FOXO proteins, thereby activating these (An et al., 2010). In the case of the p53 tumor suppressor protein a mutual regulation takes place: while mutated p53 interacts physically with VDR and changes VDR-target genes, converting 1α,25(OH)_2_D_3_ from a pro-apoptotic into an anti-apoptotic agent (Stambolsky et al., 2010), 1α,25(OH)_2_D_3_ activates the promoter of Mdm2 in a p53-dependent fashion promoting the expression of this negative regulator of p53 protein stability and function (Chen et al., 2013).

Multiple interplays between 1α,25(OH)_2_D_3_/VDR and other nuclear receptor ligands have been described. Among them, crosstalk between liganded VDR and peroxisome proliferator-activated receptor (PPAR)-α/δ in melanoma cells (Sertznig et al., 2009a,b) that may involve the stimulation of PPAR-δ expression by 1α,25(OH)_2_D_3_ (Dunlop et al., 2005). A synergistic action of 1α,25(OH)_2_D_3_ and rosiglitazone, a PPAR-γ ligand, has been shown during osteoblast-mediated mineralization (Woeckel et al., 2013a), while in human T47D breast cancer cells PPAR-γ binds VDR and represses its transcriptional activity, possibly also by competing for RXR heterodimerization (Alimirah et al., 2012). Titration out of common co-activators, but not of RXR, may be the mechanism by which ligand-bound VDR represses retinoic acid receptor (RAR) transactivation in GH4C1 pituitary cells (Jiménez-Lara and Aranda, 1999). The relation between 1α,25(OH)_2_D_3_ and retinoic acid is however complex, as cooperative effects on target genes and cellular outcome (proliferation inhibition and differentiation) have been described in other systems (Tavera-Mendoza et al., 2006; Anand et al., 2008; Ng et al., 2010). As for estrogen receptor (ER), D. Feldman's group has shown that 1α,25(OH)_2_D_3_ exerts a multilevel protective effect against breast cancer that includes the inhibition of estrogen synthesis through the direct and indirect repression of aromatase (CYP19) and the downregulation of ER-α expression through two VDREs in its promoter region (Krishnan et al., 2010; Swami et al., 2013). Likewise, there is a complex and unresolved relationship between 1α,25(OH)_2_D_3_ and androgen receptor (AR) synthesis and signaling. 1α,25(OH)_2_D_3_ induces AR in LNCaP cells (Zhao et al., 1999) while AR reduces VDR transcriptional activity (Ting et al., 2005), perhaps in some cells by a mechanism mediated by prohibitin (Mooso et al., 2010). In addition, 1α,25(OH)_2_D_3_ inhibits glucuronidation and so, inactivation of androgen in prostate cancer cells through the repression of UDP-glucuronosyltransferases (UGT) 2B15 and 2B17, which is counterintuitive given the growth promoting action of androgen and the antiproliferative effect of 1α,25(OH)_2_D_3_ in prostate cancer cells (Kaeding et al., 2008). In human bladder, 1α,25(OH)_2_D_3_ and analogs inhibit cell proliferation promoted by androgen and keratinocyte growth factor and induce apoptosis at least in part by repressing Bcl-2 expression (Crescioli et al., 2005).

Pituitary transcription factor (Pit)-1 activates growth hormone and prolactin genes in the anterior pituitary and also in breast cancer cells (Seoane and Pérez-Fernández, 2006). In MCF7 cells, VDR homodimers bind the *PIT-1* promoter and inhibit its expression in the presence of 1α,25(OH)_2_D_3_ without involvement of RXR (Seoane and Pérez-Fernández, 2006).

## Conclusions

The available evidence shows that the classical view of VDR only as a nuclear-acting ligand-modulated transcription factor that regulates the rate of transcription of those genes to which it binds is outdated. Instead, VDR and its ligand constitute a multilevel main regulator of gene expression in higher cells acting directly or indirectly, and via a variety of different mechanisms, on many signaling pathways. Some of them are triggered from the plasma membrane by paracrine or endocrine agents, and 1α,25(OH)_2_D_3_ interacts at different levels: membrane receptors, cytosolic signaling molecules or effector nuclear transcription factors. In most cases 1α,25(OH)_2_D_3_ action is mediated by nuclear VDR but in a few others this is unclear and non-canonical VDR-independent or extranuclear effects have been proposed. Available studies show that 1α,25(OH)_2_D_3_ and these signaling pathways interact variably and with distinct outcomes in a cell/tissue-specific fashion and sometimes also differentially between normal and malignant cells.

## Perspectives

The increasingly recognized importance of its non-cell autonomous actions has widened the scope of the study of 1α,25(OH)_2_D_3_. On the one hand, an in-depth study of the interplay between 1α,25(OH)_2_D_3_ and other agents, which seems to be cell-specific in terms of biological outcome, is necessary to elucidate the possibilities of combined therapies using vitamin D compounds and inhibitors or activators of a variety of signaling pathways. On the other hand, several of these interactions take place at the intercellular level. By using high-throughput techniques and genome-wide analyses, we expect to be able to identify secreted paracrine and intracellular mediators of the interaction between 1α,25(OH)_2_D_3_ and other signaling pathways responsible for the regulatory actions of 1α,25(OH)_2_D_3_ in the organism. Future research should aim to discern how vitamin D compounds modulate tissue and organ physiology and how they may be used to treat pathological processes such as infections, autoimmune disorders, or cancer.

## Author contributions

María Jesús Larriba, José Manuel González-Sancho, Félix Bonilla, and Alberto Muñoz wrote the manuscript.

### Conflict of interest statement

The authors declare that the research was conducted in the absence of any commercial or financial relationships that could be construed as a potential conflict of interest.
